# The early outcome of primary anterior sagittal approach for low anorectal malformations in female patients

**DOI:** 10.12669/pjms.36.3.1503

**Published:** 2020

**Authors:** Naima Zamir, Naima Rasool

**Affiliations:** 1 Dr. Naima Zamir, FCPS Pediatric Surgery, FCPS General Surgery, FACS. Associate Professor, Department of Pediatric Surgery National Institute of Child Health, JSMU, Rafiqee Shaheed Road, 72550, Karachi, Pakistan; 2 Dr. Naima Rasool, FCPS, FRCS, FACS, Professor of Pediatric Surgery Fazia Ruth Pfao Medical College, PAF base Mansoor and Base Faisal Hospitals, Karachi, Pakistan

**Keywords:** Anorectal malformation, Vestibular fistula, perineal fistula, Female, Child, Anterior sagittal approach

## Abstract

**Objective:**

To document our experience of the primary anterior sagittal anorectoplasty (ASARP) in female patients with lower and wide fistula in term of the early post operative outcome.

**Methods:**

A retrospective descriptive study was conducted in one surgical unit of the National Institute of Child Health Karachi, from January 2010 to April 2018. The study included female patients with diagnosis of imperforate anus with wide Vestibular or Perineal fistula with minimal or no straining during defecation and no excoriation of perineum. All patients underwent primaryASARP. Data regarding the age of the patients, site of fistula, the difficulties in dissection, post operative complications, stoma and re-do ASARP needed, were documented. Wound assessment was done during hospital stay, at two weeks and then at three months after surgery. Outcome was documented in terms of complications of surgery and cosmetic appearance of perineum. Data was analyzed on SPSS version 20.

**Results:**

A total of 70 patients underwent primary surgery, 48(68.57%) females had perineal fistula while 22(31.42%) had vestibular fistula. Age ranges between three months to 276 months with median of 6±39.73 months. No major injury to the rectal or vaginal wall occurred during surgical procedure. In early post-operative period, 12(17.14%) patients had wound infection with or without various extent of disruption. A total of seven (10.11%) patients underwent stoma formation, six (8.57%) patients because of wound disruption with in a week of primary surgery and in one patient due to severe anal stenosis and retraction of anal segment within three month follow up. Median hospital stay was 5±1.52 days. In 38(54.28%) paients complete wound healing occurred with no per or post operative complications. In 25(35.71%) patients, minor complications were noted and treated accordingly and results were labelled satisfactory with acceptable perineal appearance.

**Conclusion:**

The single stage procedure can be a good choice for both vestibular and perineal fisula. In majority of cases wound heals completely with minimal or no scaring and give good cosmetic results.

## INTRODUCTION

Anorectal malformation is a common congenital anomaly found in the pediatric population.^1^ A number of surgical procedures have been devised for the correction of the anomaly.^2^ The most common approaches currently in use are posterior sagittal anorectoplasty(PSARP) and anterior sagittal anorectoplasty (ASARP/ASAP) with or without covering stoma.^3^,^4^ In our institute, anterior sagittal approach have been in use for vestibular and perineal fistula in female patients commonly as staged procedure with good results.^5^ Nowadays surgeons are inclined toward the single stage procedure, especially in lower anomalies owing to its convenient and time saving approach.^6^ Thus avoiding multiple surgeries and the psychological impact on the child’s social behavior. After going through the literature, we revisited our approach and started doing single stage anorectoplasty. Here, we are documenting our experience of primary surgical procedure and the early outcome in female patients with vestibular or perineal fistula in term of cosmesis.

The objective was to document our experience of the primary Anterior Sagittal Approach in female patients with low and wide fistula in term of the post operative cosmetic outcome.

## METHODS

Retrospective descriptive case series was conducted in one of the surgical units of the National Institute of Child Health Karachi, from January 2010 to April 2018. Female patients with wide Vestibular or Perineal fistula with no or minimal straining during defecation and with no excoriation of perineum were included in the study. Patients were assessed for nutritional status and further evaluated for associated anomalies with ultra sound KUB and echocardiography. Informed consent from the parents was taken about the approach. Counseling regarding possible complications that may need stoma formation and re-do surgery, was done. Institutional IERB approval was taken before conducting the study. If patient was presented at neonatal age, she was kept on a regular anal dilatation with hegar’s dilator that was taught to the parents for comfortable evacuation of gut till they were subjected to primary surgery. Data regarding patient’s age and the site of fistula were noted. Preoperatively all patients were kept on an exclusively liquid diet for 48 hours before surgery, to clear liquid for 24 hours and nil per oral around six hours. All patients underwent standard anterior sagittal approach for anorectoplasty. Operative details regarding injury to the vaginal or rectal walls or any other complication were documented.

Postoperatively, all patients were kept nil per oral for 48 to 72 hours. Mothers were advised to keep the wound clean of fecal contamination by regular washing. Folly’s catheter was retained for a minimal of three days to avoid contamination with urine. Straining was prevented by avoidance of constipation. Perineal wound was assessed during hospital stay for any discharge, infection and disruption. Reassessment of wound was done after two weeks of surgery. Anal dilatation was taught to the parents. Perineal assessment was also done at one month and then at three months after surgery for the caliber and position of anal opening, and cosmetic apperence of perineum. Normally placed and well caliberated anus at proposed site with puckering around and no complications were labelled as excellent results. Normal looking anus and perineum with minor and treated complications was taken as cosmetically good results. Disruption of perineal body, retraction and stenosis of anus that need stoma formation or required redo surgery, were taken as failure of procedure. Data was documented and analyzed on SPSS version 20.

## RESULTS

A total of 70 patients fulfilled the inclusion criteria and underwent primary surgery. There were 48(68.57%) females with perineal fistula while 22(31.42%) with vestibular fistula. Age ranges between one month and 276 months with median of 6±39.71 months. Forty-seven (67.14%) patients presented and operated up till the 12 months of age, 10 (14.28%) patients were between12 to 24 months and 13 (18.57%) were above 24 months. As far as associated anomalies are concerned urological anomalies were found in 05 (7.14%) patients, while minor cardiac anomalies were found in 10 (14.28%) patients. Complex cardiac anomaly was found in one patient.

Regarding per-operative complications, no major injury to the rectum or vagina had occured during surgical procedure except small tears in initial part of anterior dissection which part was easily excised. Median hospital stay was 5±1.52 days. Post-operative results are shown in the Table-I. A total of 32/70 (45.71%) patients developed some degree of complications affecting the outcome. Superficial wound disruption was found in 12 (17.14%) patients, nine wounds were with pus discharge and three without plurent discharge. All wound infections were managed conservatively. Six patients had complete disruption of perineal body, five disruption occurred secondary to infection noted with in first week of surgery, while one patient had persistant bleeding from the wound led to disruption. All of these six patients with complete wound disruption had to undergo covering stoma before leaving hospital, while perineal wound were managed conservatively.^1^

**Table-I:** Types of fistula with post - operative complications and final outcome.

Type of fistula	Complications		Final outcomes		

			Excellent 38	Satisfactory 25	Failure 07
Perineal (48)	Superficial infection	08	0	08	0
	Wound disruption	03	0	00	03
	Rectal mucosal prolapse	02	0	02	00
	Anterior migration of anus/ minimal scarring	02	0	02	0
	Anal stenosis	01	0	01	00
Vestibular fistula (22)	Superficial infection	04	0	04	0
	Wound disruption	04	0	1	03
	Rectal mucosal prolapse	05	0	05	00
	Anterior migration of anus/ minimal scarring	01	00	01	0
	Anal stenosis	02	0	01	01
Total	70	32	00	25	07

At three months follow up, the status of anus and appearance of perineum was noted. Out of total 70 patients, 52 patients were discharged home initially without any complications. Among them, 03/70 (04.28%) patients developed anal stenosis because of non compliance of anal dilatation. They were managed successfully with one dilation under anesthesia followed by continued anal dilatations at home for next three months. In one patient, examination under anesthesia revealed severly retracted and stenosed anus for which patient underwent covering stoma followed by redo anoplasty. Seven patients developed various degrees of mucosal prolapse, only one needed mucosectomy.

In total 38/70 (54.28%), results were excellent with no complications at any stage of 03 months follow up, while satisfactory results were noted in 25/70 (35.71%) patients with minor complications which were dealt accordingly. (Mucosal prolapse seven, vaginal and rectal tear, superficial wound infections 12, anal stenosis three, and mild anterior migration of anus three).

Failure of one stage surgery was considered in 07/70 (10.11%) patients when covering stoma was needed followed by redo surgery, six for pernieal body disruption and one for retracted rectum and stenosed anus.

## DISCUSSION

Owing to the better understanding of embryology, anatomy and physiology of perineal muscle complex, and general improvement in the neonatal care made it possible to manage the vestibular and anal fistula in female babies in one stage.^1^ This is precluding the need of stoma formation, which has cumbersome impact on socio psychosocial behavior of the patient. The good outcome of single stage surgery reported in literature significantly reduces the hospital workload and putting less burden on family.^7^,^8^

Performing Primary surgery in newborn baby is technically easier due to shorter distance between rectum and skin, thus less tension in anorectal anastomosis. There are better chances of continence and toilet training is easy which is making surgeons incline to do primary ASARP in newborns.^9^,^10^ Unlike this fact, in current data, we have patients presented at much later age possibly because of the fact that vestibular or perineal fistulas with imperforate anus are oftenly missed in the neonatal life due to presence of meconium and then stool at the perineum especially with wide fistula. Problem is usually identified when there is either straining or constipation or noticed by caretaker at home, while cleaning of perineum.^11^,^12^ Perhaps due to this, some girls do report at later age in our study, as one patient presented at 23 years of age, with passage of stool from vestibular opening and absence of anus.

Although primary surgery is now days performed in neonatal age with good results, but since the neonatal post-operative care is still lacking in resource - restrained countries like Pakistan, we prefer to do elective surgery at a minimum of 2-3 months. Until then the patient is advised for regular anal dilatation to keep the gut decompressed.

Selection of the patient for primary procedure is very important to prevent complications, which may necessitate stoma formation or re- do ASARP. However, one of our patient of ARM with rectovestibular fistular, who also had Tetralogy of Fallot for which surgery was awaited. She exhibits straining during the defecation, which is considered a relative contraindication for one stage surgery due to increased tendency of wound disruption due of straining. To avoid multiple surgeries and trauma of anesthesia, she was subjected to one stage surgical procedure. With austere preoperative preparations, per operative vigilance and astute postoperative care, the outcome was excellent, which indicates that primary anorectoplasty can be a good option in such critical patients.

The commonest complications which urge stoma formation and eventually necessitates re do ASARP are wound infection with complete wound disruption, anal stenosis and retraction of anal canal and recurrent rectovestibular fistula.^7^,^13^,^14^ To prevent these complications, every step should be taken to optimize the patient for preoperative preparation, gentle tissue handling during surgery, meticulous hemostasis, and avoidance of injury to vaginal or rectal wall during dissection, adequate mobilization of the ano- rectal segment and tension free anastomosis at anal verge. Special attention ought to be given to the wound care in early post-operative period along with Postoperative antibiotics coverage and retaining urethral catheter for three days to avoid the contamination of wound with urine to minimize the wound infection.^15^,^16^ However, in our study, per operative tear of vaginal or rectal or both walls occurred in 27/70 (38.57%) of patients in the initial part of dissection, which is at par of other studies. The torn tissue was excised convieniently and did not have any effect on the outcome of surgery.

As far as superficial wound infection is concerned, it was noted in 12/70 (17.14%) of patients. These were managed with conservative treatment and did not lead to any other additional procedure. While in 06/70 patients (8.57%), deep wound infection and complete wound disruption, led to stoma formation and subsequent re do ASARP. In our study, slightly increased rate of wound infection as compared to current literature is perhaps because of late presentation of patients leading to hypertrophy of rectal wall and fecaloma formation, which may result in wound contamination during or after surgery 16,17 One patient who had significant rectal mucosal prolapse, which had to be excised surgically, while no recurrence occurred in our series.

A total 04/70 (05.71%) patients developed anal stenosis. Three were due to noncompliance of post-operative anal dilatation, managed conservatively with first anal dilatation under general anesthesia followed by further anal dilatation at home, while one patient had intractable stenosis ended up in staged repair. This constitutes 07/70 (10.00%) of our failure cases of one staged procedure. This seems at par to other studies reported like Gupta et al and Wakhlu et al in 2017 and 2009 respectively.^16^-^17^

Apart from anatomical location of neo anus, assessment of physiological consequence is key factor in the postoperative outcome of anorectal malformations but the issues of soiling, constipation and incontinence are not frequently reported in patients with rectovestibular or perineal fistula because of following reasons. First, in lower anomalies, the rectal pouch has already passed through the sphincter complex and second, in the ASARP, the sphincter is cut in anterior aspect only and the rectal pouch is placed in the center of sphincter leading to sphincter preservation.^17^-^19^ In long-term follow up, adequate and strong perineal body between the rectum and vagina is of great importance for prevention of recurrent urinary tract infection, damage perineum and recurrent rectovaginal fistula in adult life.^20^

## CONCLUSION

The single stage procedure can be a safe and beneficial choice in female patients for both vestibular and perineal fistula in selected cases. Appropriate selection and preparation of patients, vigilant surgery, and needful post-operative care of wound can give excellent cosmetic results and prevent failure of procedure and decreases the burden on treating surgeon, family and the patients.
